# Assessment of Major Causes of Calf Mortality in Urban and Periurban Dairy Production System of Ethiopia

**DOI:** 10.1155/2020/3075429

**Published:** 2020-02-24

**Authors:** Tsegaw Fentie, Sintayehu Guta, Gebreyes Mekonen, Wudu Temesgen, Achenef Melaku, Getachew Asefa, Shimelis Tesfaye, Ayalew Niguse, Bosenu Abera, Fikre Zeru Kflewahd, Birhanu Hailu, Feyissa Begna, Zemene Worku

**Affiliations:** ^1^University of Gondar, College of Veterinary Medicine and Animal Sciences, P. O. Box 196, Gondar, Ethiopia; ^2^National Animal Health Diagnostic and Investigation Centre, P. O. Box 04, Sebeta, Ethiopia; ^3^Bahir Dar Regional Veterinary Laboratory, P. O. Box 79, Bahir Dar, Ethiopia; ^4^Jigjiga University College of Veterinary Medicine, P. O. Box, Jigjiga, 1020, Ethiopia; ^5^Samara University College of Veterinary Medicine, P. O. Box 132, Semera, Ethiopia; ^6^Jimma University College of Agriculture and Veterinary Medicine, P. O. Box 307, Jimma, Ethiopia

## Abstract

A cross-sectional calf mortality study was conducted in urban and periurban dairy farms in Addis Ababa, special zones of Oromia and Amhara regions in July and August 2015. The objectives of the study were to estimate the annual mortality and to assess the major causes of calf mortality in the dairy farms. One-year retrospective data on calf mortality were collected from 330 farms by face-to-face interview using the pretested and structured questionnaire format and direct observation of farm practices. A logistic regression analysis was performed in order to identify the predictor variables associated with early calf mortality. Data were analysed using Statistical Package, Stata SE for Windows, version 12.0. The annual mean calf mortality from birth-to-weaning was reported as 18.5% (95% CI: 12.6, 24.3%). The prenatal loss due to fetal death and stillbirth was 10.1% (95% CI: 6.7, 13.6%). The overall annual loss due to fetal death and calf preweaning mortality was 26.7% (95% CI: 21.2, 32.2%). Age-specific mortality declined with increased age, and the highest mortality was recorded during the first month of life extending up to the third month of age. Disease was the most important causes of calf mortality (73.2%). Among the diseases, diarrhea (63%) and respiratory disorders (17%) were the important causes of calf mortality. Malpractices in calf management were identified, including restricted colostrum and milk feeding, poor care and supplemental feeding, and poor health management. Interventions in dairy cattle health and farm husbandry are recommended to control calf mortality.

## 1. Introduction

Ethiopia holds a huge potential for dairy cattle development. The development of the dairy sector in Ethiopia has considerable prospective opportunity for smallholder employment and income generation and may contribute significantly to poverty alleviation and food and nutrition security. However, livestock production and productivity is low because of poor genetic potential of indigenous breeds, frequent seasonal drought, feed shortage in quantity and quality, high prevalence of rampant animal diseases, and poor infrastructure and animal health services [1].

Calf mortality is considered as one of the major constraints to herd expansion and genetic improvement in the dairy sector. The calf morbidity and mortality studies in Africa indicate high calf loss both in the subsistence and market-oriented dairy production systems. Studies of calf mortality on smallholder farms indicate preweaning and early postweaning mortality rates in the range of 15% to 25%. In some African countries, for instance in Tanzania, calf mortality rates range from 9% to 45% [2, 3], and in Mali, the range is 10% to 25% [4]. In Sudan, 4.9% mortality was reported in dairy farms in Khartoum [5]. In Ethiopia, 30% preweaning calf mortality rate was reported in mixed crop-livestock production systems in the Amhara region [6], and 18% mortality rate was reported in market-oriented dairy farms in Central Ethiopia [7]. Neonatal calf mortality is the most important constraint in young stock and accounted for 8.7 to 84% of the total calf mortality [8].

Both infectious and noninfectious causes contributed to the morbidity and mortality of the calf. Calf diseases that cause morbidity and mortality are the results of the complex interaction of the management practices, the environment, infectious agents, and the animal itself. Mortality of neonates was mainly attributed to conditions such as diarrhea and pneumonia associated with poor housing, hygiene, and nutrition [9]. Different management and environmental factors such as colostral feeding, housing, calving assistance, production system, herd size, season, and hygiene of microenvironment were reported to affect significantly calf morbidity and mortality. It is estimated that 20% calf mortality resulted in 38% reduction in the profit of a livestock farm [10].

Experience indicated that young animal losses can be significantly reduced by introducing new techniques of management, including proper feeding and nutrition, housing, and hygiene [11]. Furthermore, herd replacement can be attained by improved management strategies that can reduce stillbirth and preweaning mortalities. Improved management strategies can increase the number of replacement stock in the herd from 15% to over 35% and will allow farmers to increase their herd sizes [8].

The current Ethiopian livestock breeding policy emphasizes upgrading the genetic makeup of the local stock through crossing with high-grade exotic breeds of cattle. As a result, the proportion of crossbred calves is gradually increasing in the smallholder dairy farms mainly in the highlands of the country, suggesting a susceptible population that will need improved health and proper management. One of the major health and management intervention areas recently proposed in the Livestock Development Master Plan is aimed at reducing young and adult stock mortality [12].

Efficient production and limited losses are important for the livestock producers to realize benefits from their livestock resource. In order to minimize losses, the causes of animal morbidity and mortality and the associated risk factors need to be identified, and appropriate control measures are implemented. This study was undertaken with the interests of the livestock ministry to generate information on calf mortality for designing and implementing control strategies.

## 2. Materials and Methods

### 2.1. Study Area and Animals

**Figure 1:**
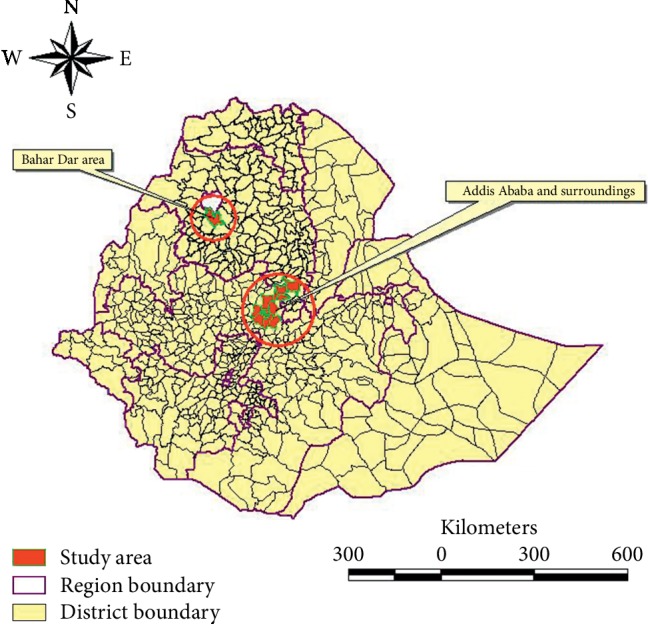
Map of study sites.

This study was conducted in the urban and periurban dairy production system, located in the milk sheds of Addis Ababa and surrounding districts of Oromia (i.e., Sebeta, Sululta, Holleta, Bishoftu, and Sendafa towns) and districts of the Amhara region (Chacha, Debre Birhan, and Bahir Dar) as shown in Figure 1. The study animals consisted of Holstein Friesian crossbred cattle targeted mainly for milk production. Calf in the context of this study includes young calves born alive and those below one year of age. Premature fetus born dead during the last trimester of pregnancy was considered as prenatal loss of calf. The age of calves was estimated by the enumerators based on available birth records and dentition for the calf which will develop 8 temporary incisors by the first month of age.

### 2.2. Study Design

A cross-sectional study design was employed during July and August 2015 in selected dairy farms. Survey was conducted to estimate annual calf mortality and to identify causes of mortality in the study population.

### 2.3. Sample Size and Sampling

Sample size was determined assuming that 70% of dairy cattle farmers have adequate knowledge about possible causes of calf mortality and farm management practices and can properly respond to the interviews at 95% level of confidence and 0.05 desired precision. The relevant sample size formula provided by Dohoo et al. [13] was used:

(1) $$\begin{array}{c} n=\frac{Z_{\alpha }^{2}\cdot pq}{L^{2}}, \end{array}$$

where *n* = sample size, *Z*_*α*_ = the value of standard normal distribution at 95% level of confidence (Z_*α*_ = 1.96), *p* = expected proportion (70%), *q* *=* 1 − *p*, and *L* = the required absolute precision of the estimate.

Accordingly, the calculated sample size was 323 household farms, but to make the distribution proportional, 330 farms were identified (i.e., 110 farms per region) for sampling. The sample size was then proportionally distributed among regions and production areas (urban and periurban).

Stratified sampling was used to categorize the study dairy population by region and production areas (urban and periurban). Farms were sampling units, and the list of dairy farms was constructed in collaboration with livestock development agents in the districts. Then, study farms were selected randomly but with some restrictions on the selection imposed based on the willingness of farm owner's to participate in the study.

### 2.4. Data Collection and Analysis

A structured and pretested questionnaire format was used to collect data from farm owners or animal attendants. Trained enumerators were used to collect one-year retrospective data on herd structure, calf morbidity and mortality, causes of mortality, and farm management practices such as assistance during parturition, calf colostrums, and milk-feeding practices. The standard young stock management was used to assess the farmers' knowledge and practices. Clinical and body condition examination was made for calves less than one year old to assess individual calf health status. A checklist of parameters was also used to evaluate the feeding and housing conditions during farm visit.

Descriptive and analytical statistics was used to analyze data using Stata statistical software. Causes of calf mortality were compiled, and percentage contribution (proportion) of each cause for mortality was calculated and presented.

Mean annual birth-to-weaning calf mortality was determined by dividing the number of deaths by the number of alive births within a particular study year. The overall annual calf loss was calculated by dividing the total prenatal (fetal) death and birth-to-weaning deaths by expected births. Prenatal death in terms of abortion and stillbirth during the same study year was also calculated (number of stillbirths divided by expected births (number of abortions and stillbirths + alive births)). Mean annual mortality was calculated at aggregated level with 95% confidence intervals assuming that the study population was normally distributed, sample size was large enough, and outliers were minimal and, if any, were omitted from the analysis.

Logistic regression analysis was performed to identify risk factors for calf mortality. The factors related to calf birth situations, time, and management were considered as independent variables and calf death as dependent/outcome variable, and demographic factors were considered as independent and knowledge or practice as an outcome variables. The end results of the analyses were final models including all variables (risk factors) significantly associated with calf mortality. The models were described in terms of odds ratios with 95% confidence intervals.

## 3. Results

### 3.1. Herd Structure and Size

The mean herd size of the study farms in the urban and periurban dairy system was 14.7 heads (95% CI: 12.5–17.0). A total of 330 farms consisting of 4,898 heads of cattle were studied. The largest herd size was in Oromia region, 18.2 (95% CI: 14.3–22.2), followed by Addis Ababa, 14.2 (95% CI: 11.9–16.4), and 10.5 (95% CI: 5.6–15.5) in the Amhara region. All the farms consisted of Holstein Friesian crossbred cows targeted mainly for milk production. The proportion of cows was about 55.8%, and the proportion of heifers and calves was 24.0% and 20.8%, respectively. Urban and periurban dairy production is a recently growing livestock subsector and a means of family income and livelihood for some smallholder farmers. In the present study, it was reported that dairy production is a primary business for 59.3% (*n* = 197) of farm owners.

### 3.2. Estimated Calf Mortality

The calf mortality in the urban and periurban dairy production system was skewed; 41.4% of farms recorded calf mortality. Prenatal and birth-to-weaning mortality in urban and periurban dairy farms in the different regions is summarized in Table 1. The overall calf loss due to prenatal (abortion/stillbirth) and birth-to-weaning was estimated 26.7% (95% CI: 21.2, 32.2) of which 10.1% (6.7, 13.6) was due to prenatal loss and 18.5% (95% CI:12.6, 24.3) due to birth-to-weaning mortalities. The mean annual birth-to-weaning mortality was significantly higher in the Addis Ababa region, 25.5 (95% CI: 10.0, 40.9%) compared to Oromia, 15.3 (95% CI: 10.5, 20.2%), and Amhara Region, 14.9 (95% CI: 8.7, 21%).

The prenatal mortality in terms of abortion and stillbirth during the study year was relatively higher in Oromia, 14.4% (95% CI: 7.2, 21.6), while reports were found to be similar in Addis Ababa, 6.9% (95% CI: 4.1, 9.7) and Amhara region, 6.3% (95% CI: 3.7, 8.9). Health status and parity stage of the dam, big fetus size, and difficulty in birth (dystocia) were some of the reasons mentioned by farmers as causes of stillbirths.

Mortality was inversely related to age of calves. A higher mortality rate was reported at lower age groups in neonates and decreased with increased age. Early mortality for live-born calves during the first one month of life accounted for 64.2% of the total mortality and was particularly high during the third week of life.

### 3.3. Causes of Calf Mortality

Among the causes of calf mortality recognized by the farmers during the individual interview, disease was the major problem, followed by sudden deaths and accidents (Table 2). The mean annual mortality owing to disease was 13.4% (7.8, 19.0). The contribution of disease to calf mortality was higher in Addis Ababa (54%) and Oromia regions (33.5%). Death of small and weak calves at birth was also considered important as these animals need frequent attention and support, but owners may actually not provide this extra support. Sudden deaths were deaths with unrecognized syndromes. Malnutrition was also reported as one of the problems. Hand feeding is practiced by a portion of the dairy farmers, and some of the farmers give less attention to feeding of male calves.

Of 229 disease syndromes recorded prior to deaths of calves (Table 3), the mean mortality attributable to diarrhea was the largest (63%), followed by respiratory disorders. Gastrointestinal problems causing diarrhea were very common in neonates, in the first one month of life. Considerable mortality was reported related to general disease syndromes (nonspecific), such as shivering, anorexia, and sudden deaths. Lumpy skin disease was reported from a few farms that faced outbreaks during the study year.

### 3.4. Effects of Farm Management Practices on Calf Mortality

Some common farm management practices and host-related variables were assessed for their impact on calf mortality. Navel treatment practice and colostrums/milk feeding method were identified as risk factors for mortality of calves (Table 4). Significantly higher mean mortality was reported in farms that practice hand feeding of colostrum and milk and in those that did not practice navel treatment. All other management variables showed no statistically significant (*P* > 0.05) effect on mortality. However, it was noted that the odds of mortality was >1 in dam parity and herd size, perhaps due to wide differences in mortality rates among categories.

## 4. Discussion

A high calf mortality that reaches up to a loss of a quarter of calves was observed in the study urban and periurban dairy farms in Ethiopia. The birth-to-weaning mortality rate reported in this study (18.5%) was consistent with the previous calf mortality reports in the tropics of Asia and Africa, which is in the range of 15–25% [14]. It was also comparable to previous calf mortality studies in Ethiopia, 18% calf mortality was reported in Debre Zeit [7] and Bahir Dar milk sheds [6]. This rate is very high as compared to calf mortality rates in developed dairy farming in the temperate world. For example, a calf mortality of less than 6% in the UK [15] and 3% in Australia [16] is achieved through better calf management.

The mortality rate was seen to vary by age category, the highest being in the first one month of age (9.6%). Generally, more than 64% of calf mortality occurred within one month of age, and about 50% mortality occurred in the first week of life. Important calf diseases such as calf scour occur in the early life of the calf, and this could be one of the reasons for high calf mortality in the first month of life. Calves may also be vulnerable to environmental stress at an early age. The high mortality of calves during the first month of age suggests that there should be given more attention to calf management in the first few weeks of life.

High calf mortality seriously affects the business of the dairy farms. Normally, 20–25% of cows are expected to be replaced annually in dairy farms [14]. A loss of 18.5% reported in the present study means the farmers cannot raise enough stock to replace loss, let alone expand the herds. Due to less-developed milk processing and marketing in Ethiopia, the income from milk sales for most smallholder dairy farmers is not reliable, and most of them consider sales of heifer an important source of farm income. As such, high calf mortality is a very important problem for the businesses of smallholder urban and periurban dairy farmers in Ethiopia.

Prenatal loss in terms of stillbirth reported in this study (10.1%) is also an important challenge for the dairy development. An excellent calf management program begins with care of the dam prior to calving. Reports showed that about 11–13% of calves born to first lactation dams are stillborn, while calves born to older dams are half as likely to be stillborn [17]. Calf mortality is influenced by the health of the dam. Additionally, calves have a better chance of survival if stress during the birth process is minimized. Key factors that influence stress include size of calf, health of dam, crowding, and cleanliness of the calving environment and quality of assistance provided [17].

Based on the farmers' observations, disease was the major cause of mortality in the dairy production system of the study area. In the present study, the mean calf mortality attributed to diseases is 13.4%, and the contribution to the overall mortality is 73.2%. This indicates that the most important areas of intervention in reducing calf mortality should be health management, which may include proper passive immunity transfer and biosecurity. Calves at born would not have specific immunity, and optimum passive immunity through proper colostrum feeding needs to be ensured to increase resistance of calves to early infections. Studies showed early ingestion of colostrum reduces calf morbidity [7]. Good hygiene in calf pens was also seen to be associated with reduced calf morbidity and hence may reduce calf mortality.

Among disease conditions, calf diarrhea was noted as the first disease problem of calves, followed by respiratory problems. Mortality as a result of diarrhea was 63% (46.4, 79.4) and 17% (was related to respiratory problems). The major causes of calf mortality reported worldwide are diarrhea (calf scours) and respiratory diseases [4, 18]. Previous studies in Ethiopia, which involved a longitudinal follow-up, also showed diarrhea as the first important disease of calves [6, 7], and its prevalence appears to be management-related, especially when calves are housed in unhygienic conditions [7]. These conditions can be controlled with significant reduction in calf mortality by observing good calf management that includes adequate colostrum intake soon after birth, good housing, and well-managed healthy dams.

High calf mortality is believed to be due to less attention given to, and limited resources devoted to, calf management by farmers because there is no immediate income derived from calves [14]. Good calf rearing is important as it ensures availability of good future replacement stock. In this study, calves in most smallholder dairy farms are not performing well. Apart from that, farmers aim to optimize income by selling more milk and calves are, therefore, underfed. So, intervention focusing on calf management could decrease the problem. Extension programs related to calf management were observed to decrease the high calf mortality in the tropics, e.g., in Kenya [19] and Sri Lanka [20].

Most farmers do not have enough knowledge of proper calf-feeding regimes. Farmers provided their calves with poor-quality feed, mainly natural grass and dry crop residues. The nutritional value (crude protein, mineral contents, and digestibility) of these feed stuffs has been found to be lower than that of legumes [21]. There was a scarcity of protein as there was no farmer who fed protein supplements to the calves during the study period. Poor calf body conditions observed during farm visits of the present study could be the impact of this poor nutrition. However, crop residues can be utilized more efficiently by offering them to animals along with urea molasses blocks, which have shown good results [22].

In logistic regression analysis of different management-related factors, navel treatment practice and milk/colostrum feeding methods were identified as risk factors for mortality of calves. High mean mortality (12.7%)was reported in farms that practice a hand-feeding method of colostrum and milk. Besides, high mortality was reported in farms that did not practice navel treatment (13.6%). Hence, calf suckling and navel treatment can significantly (*P* < 0.05) reduce mortality of calf in the study area. All other variables had no statistically significant effect on mortality. Compared to hand feeding, suckling is a greater source of absorption of colostral immunoglobulins; therefore, it is generally recommended to allow calf to suckle its mother for the first two days postpartum [23]. Mortality can also be reduced by navel disinfection and by improving housing conditions.

## 5. Conclusions

Urban and periurban dairy production is a recently growing livestock subsector and a means of family income and livelihood for smallholder farmers. In the present study, it was reported that dairy production is a primary business for many of farm owners. The development of the market-oriented dairy sector in Ethiopia has considerable potential for smallholder employment and income generation and may contribute significantly to poverty alleviation and food and nutrition security. However, preweaning mortality of calf appeared to be one of the major constraints in the study farms, hampering the development of replacement stock. The critical time for higher calf mortality in this production system was during the first one month of life extending up to the third month of age.

Disease and malnutrition appeared to be the most important causes of calf mortality. Among diseases, diarrhea and respiratory infections were the most common challenges of raising calves. The study also revealed malpractices in calf management among the studied producers, including poor management, restricted colostrum feeding, and poor care, especially for the calves in terms of milk allowance, supplemental feeding, and health management. While many herders appeared to be aware of the challenges of raising calves, they seemed to have limited knowledge to deal with the challenges. However, many of the health problems of calves can be controlled by excellent early nutrition and management.

## Figures and Tables

**Table 1:** Mean annual calf mortality and distribution by age category in urban and periurban dairy production system, 2014 to 2015.

Age category	Mortality (%) (95% CI)
Prenatal mortality (abortion and stillbirth)	10.1 (6.7, 13.6)
Perinatal (<48 hrs)	2.4 (1.8, 3.3)
Neonatal (48 hours–1 month)	9.6 (8.3, 11.1)
Early preweaning (1–3 months)	2.2 (1.3, 3.0)
Late preweaning (>3 months)	1.5 (1.0, 2.2)
Mean annual birth-to-weaning mortality^*∗*^	18.5 (12.6, 24.3)
Overall mean annual mortality^*∗∗*^	26.7 (21.2, 32.2)

^*∗*^Mean birth-to-weaning mortality calculated related to total number of calf born alive (*n* = 1,706). ^*∗∗*^Overall annual mortality calculated related to total number of calf born alive + stillborn (*n* = 1,898).

**Table 2:** Causes of calf mortality in the dairy production system.

Cause of mortality	Relative contribution of causes, proportion, *n* (%)^a^	Cause-specific mortality (95% CI)^b^
Disease	229 (73.2)	13.4 (7.8, 19.0)

Small and weak at birth	18 (5.8)	1.1 (0.5, 1.5)

Bloating	11 (3.5)	0.7 (0.3, 1.0)

Accidents and sudden deaths	29 (9.3)	1.7 (1.1, 2.3)

Dystocia	6 (1.9)	0.4 (0.1, 0.6)

Malnutrition	12 (3.8)	0.7 (0.3, 1.1)

^a^Each cause expressed as number of calve deaths due to the specific cause relative to the number of deaths (*n* = 313). ^b^Calculated calf mortality rate due to each cause relative to the number of alive born (*n* = 1706).

**Table 3:** Disease syndromes related to calf mortality in dairy production system (*n* = 224).

Disease/syndrome	Mean mortality (%) (95% CI)
Diarrhea	63.0 (46.4, 79.4)
Respiratory	17.0 (9.4, 24.5)
Lumpy skin disease	4.9 (1.3, 8.5)
Bloat	2.2 (0.1, 4.6)
Nonspecific	12.1 (4.1, 19.6)

**Table 4:** Logistic regression analysis of management-related risk factors for mortality of calves in the dairy production system.

Variables	Category	Mortality rate *n*(%)	Odds ratio (OR)	95% CI (OR)	*P* value
Time of birth	Day	193 (11.3)	1.0		
	Night	116 (6.8)	0.95	0.60, 1.52	0.835

Delivery status	Normal	107 (6.3)	1.0		
	Assisted	9 (0.5)	1.05	0.60, 1.98	0.886

Dam parity	Primiparous	107 (6.3)	1.0		
	Multiparous	194 (11.4)	1.08	0.60, 1.81	0.775

Herd size	1–10	96 (5.6)	1.0		
	10–20	61 (3.6)	1.13	0.63, 2.02	0.686
	20–30	112 (6.6)	1.63	0.83, 3.18	0.157
	>30	40 (2.3)	1.94	0.65, 5.78	0.236

Calving facility	Yes	37 (2.2)	1.0		
	No	272 (16.0)	0.76	0.3, 1.61	0.470

Navel treatment	Practiced	77 (4.5)	1.0		
	Not practiced	232 (13.6)	0.41	0.23, 0.73	0.003^*∗*^

Colostrum feeding	Full suckling	301 (17.7)	1.0		
	Restricted	8 (0.5)	0.56	0.20, 1.53	0.256

Colostrum and milk feeding method	Hand feeding	216 (12.7)	1.0		
	Suckling	93 (5.5)	0.48	0.28, 0.83	0.008^*∗*^

^*∗*^Significantly different (*P* < 0.05).

## Data Availability

Data were collected through a questionnaire survey on calf mortality in urban and periurban dairy farms of Ethiopia. Epidemiological questionnaire survey focused on husbandry, disease prevention and control, housing and feeding, and other relevant farm management practices. The data used to support the findings of this study are available from the corresponding author upon request.
